# Editorial: Methods and Applications in Molecular Phylogenetics

**DOI:** 10.3389/fgene.2022.923409

**Published:** 2022-07-14

**Authors:** Juan Wang

**Affiliations:** School of Computer Science, Inner Mongolia University, Hohhot, China

**Keywords:** molecular phylogenetics, whole genome sequences, protein, application, disease

The purpose of molecular phylogenetics is to infer the evolutionary history of organisms and gene sequences. In the early stages of research, molecular phylogenetics mainly considers the changes vertically, such as insertion, substitution, and deletion in loci (Siepel and Haussler, 2004). With the development of sequencing technologies, the whole genomes are available for more and more organisms and are used to analyze their phylogenetics (Henz et al., 2005; Birin et al., 2008). The evolutionary history of organisms at this stage is described as a phylogenetic tree (Bruno et al., 2000). Then, genes of genomes are rearranged under horizontal events, such as inversions, duplications, and transpositions, which change the content and order of genes. Many studies introduce computing methods of molecular phylogenetics for whole genomes (Greenman et al., 2012). Phylogenetic networks are used to describe the evolutionary history (Wang and Guo, 2019). Molecular phylogenetics has been applied in many areas, such as the analysis of proteins (Lv et al., 2020).

Traditional methods for molecular phylogenetics need to do the alignment for sequences. It is very time-consuming to process the alignment of whole genome sequences. Therefore, it is a hard issue to do phylogenetic analysis from whole genome sequences of organisms. Wu et al. introduce a metric called information-entropy position-weighted k-mer relative measure (IEPWRMkmer), which combines the position-weighted measure and the information entropy of frequency for k-mers. Accordingly, they denote the whole genomes as feature sequences and then use Manhattan distance to compute the distance between two whole genomes. Finally, they use the Neighbor-Joining method to construct the phylogenetic tree from distance matrices. The IEPWRMkmer is efficient and effective for extracting key information for evolutionary analysis, and it is free to align for whole genomes.

Many studies have been done in applications of molecular phylogenetics. A protein complex contains proteins that interact with each other in function due to the evolutionary relationship. Wang et al. used semantic information of GO terms and the topological information of PPI networks to propose a method called TSSN for constructing a weighted PPI network. They proposed a new algorithm (NNP) for recognizing protein complexes from the weighted PPI network. Experiments showed that the algorithm could identify more protein complexes more accurately. PredMHC, proposed by Chen et al., is used to predict major histocompatibility complex (MHC). The PredMHC extracts information on amino acid composition from proteins, which is different due to the evolution of coding genes. It uses the voting of the SGD, the SMO, and random forest to predict and achieve the best performance on both training and testing datasets than other methods.

Molecular phylogenetics is also applied in predicting disease-related proteins. Anti-inflammatory peptides (AIPs) are important to treat some inflammatory and autoimmune diseases. Zhao et al. introduced a model (called iAIPs) to identify AIPs. iAIPs extract features from AIPs based on the information of sequences changed in evolution and then use the random forest to train. Experimental results show that iAIPs can identify AIPs accurately. Cancer is a serious threat to human health and is one of the main causes of disease death. MultiGATAE, proposed by Zhang et al., can identify the cancer subtypes. It first constructs a similarity graph from multi-omics data (i.e., mRNA, miRNA, and DNA methylation) and then uses a deep learning method to learn embedding representation. It uses the K-means clustering method to identify cancer subtypes from embedding representation.
